# Acclimation Effects of Natural Daily Temperature Variation on Longevity, Fecundity, and Thermal Tolerance of the Diamondback Moth (*Plutella xylostella*)

**DOI:** 10.3390/insects13040309

**Published:** 2022-03-22

**Authors:** Kun Xing, Fei Zhao

**Affiliations:** Shanxi Key Laboratory of Integrated Pest Management in Agriculture, College of Plant Protection, Shanxi Agricultural University, Taiyuan 030031, China; xingkun@sxau.edu.cn

**Keywords:** thermal acclimation, temperature amplitude, life history trait, thermal tolerance

## Abstract

**Simple Summary:**

Diurnal, monthly, or seasonal temperatures can fluctuate substantially. Daily temperature amplitudes (DTAs) can significantly impact the traits of insects but there is limited evidence from the natural environment. Therefore, we studied the acclimation effects of DTA on the longevity, total fecundity, early fecundity, and thermal tolerance of adult diamondback moths (*Plutella xylostella*) under environmental conditions. The longevity, total fecundity, early fecundity, and heat thermal tolerance of adults significantly changed under different DTAs. These findings highlight the effects of DTA on the acclimation response in the *P.* *xylostella* phenotype, and DTA should be incorporated into prediction models for assessing insect populations and the effects of climate change.

**Abstract:**

Daily temperature amplitudes (DTAs) significantly affect the ecological and physiological traits of insects. Most studies in this field are based on laboratory experiments, while there is limited research on the effects of changes in DTA on insect phenotypic plasticity under natural conditions. Therefore, we studied the acclimation effects of DTA on the longevity, total fecundity, early fecundity, and the thermal tolerance of adult diamondback moths (*Plutella xylostella* L.) under naturally occurring environmental conditions. As DTAs increased, male longevity and total fecundity decreased, and early fecundity increased. An increase in DTA was significantly associated with the increased heat coma temperature (CT_max_) of both males and females, but had no significant effect on their cold coma temperature (CT_min_). Our findings highlight the effects of DTA on the acclimation response of *P. xylostella* and emphasize the importance of considering DTA in predicting models for assessing insect populations and the effects of climate change.

## 1. Introduction

Temperature has a significant effect on the physiological metabolism [1], growth and development [2,3], survival and reproduction [4,5,6], behavior [7,8], and other core life activities of organisms. Environmental temperatures not only vary in terms of mean temperature but also fluctuate daily, monthly, and seasonally [9]. Many researchers have focused on the effects of climate change on biological phenotypes under different mean temperatures in recent decades [10,11]. In recent years, the effect of temperature fluctuations on different organisms have attracted increasing attention [12,13,14].

Acclimation is a process in which organisms adjust to a change in their environment, such as atmospheric pressure, temperature, humidity, photoperiod, and pH. Temperature acclimation has an important effect on various organisms [15] such as mollusks [16], reptiles [17], birds [18], and fish [19], and it also has a significant effect on insects, which have a short life cycle and high sensitivity to temperature [20]. Temperature acclimation substantially affects the prospects for insect population dynamics and survival [21]. Laboratory studies have mainly focused on constant temperatures [22], while fewer studies have investigated two-step temperature variation [23,24,25] or only simulated daily high-temperature changes to investigate the responses of insect life history traits [26], heat tolerance [27], thermal escape behaviors [28], and fitness [29]. In the field, some studies have explored the mixed effect of acclimation to the environmental mean and variance in temperature on insect behavior [30], life-history traits [31], physiological and biochemical traits [32], temperature tolerance [33], and gene expression [34]. To improve ecological relevance, the effects of temperature acclimation should pay more attention to temperature amplitudes at a similar mean temperature. There is a lack of studies investigating thermal tolerance and the underlying functional transcriptomic responses in relation to insect acclimation to daily temperature amplitude [35].

*Plutella xylostella* L. (Lepidoptera: Plutellidae) is a major pest of cruciferous vegetables and it is distributed worldwide [36]. Studies have shown that temperature has a significant effect on not only the development [37], survival [38], and reproduction [39] of *P. xylostella* but also its migration pattern and regional distribution [40]. Correlation studies between *P. xylostella* traits and temperature have mainly focused on the changes in a constant temperature gradient or on a two-step temperature change [41,42]. The amplitude of temperature change has significant effects on the different developmental stages of *P*. *xylostella* in the laboratory [43,44,45]. The acclimation effects of natural temperature fluctuations on the life-history traits and temperature tolerance of *P*. *xylostella* need further verification.

Therefore, in this study, we examined the influence of temperature amplitudes under environmental conditions on the phenotype of *P*. *xylostella*, including adult longevity, total fecundity, early fecundity, and heat and cold tolerance. We addressed the two following questions: (1) what are the acclimation effects of natural temperature amplitudes on longevity, total fecundity, early fecundity, and other traits? (2) Do natural temperature amplitudes affect temperature tolerance?

## 2. Materials and Methods

### 2.1. Insect Rearing

*Plutella xylostella* individuals were collected from Wuhan, China (30.62° N, 114.13° E), and were raised indoors on an artificial diet (Southland Products, Inc., Lake Village, AK, USA) at a constant temperature of 25 ± 1 °C, relative humidity (RH) of 50–70%, and a daily cycle of 15 h of light and 9 h of darkness [45].

A 24-well orifice plate was sterilized by irradiation with ultraviolet light for 30 min. Thereafter, 1.7 mL of the artificial feed was injected into each well to feed *P. xylostella*. New eggs (laid within the last 4 h) were transferred to each well (one egg per well) using a paintbrush. The perforated plates were covered with a fine (200 mesh) nylon mesh for ventilation and placed outside at an ambient temperature. The artificial feed was replaced with fresh feed once daily. The development and survival of *P. xylostella* individuals were recorded at 08:00 h every day. Within 4 h of pupal emergence, the adults were moved indoors to mate and breed. Newly paired adults were moved to a glass tube (3 cm × 12 cm) for mating and spawning. A nylon mesh (200 mesh) was attached to both ends of the glass tube, and cotton balls (immersed in 10% honey water) were placed in the tubes for feeding. From 15:00 to 16:00 h, the egg cards (3 cm × 4 cm sealing film soaked in cabbage juice) were placed in each glass tube for egg laying. The glass tubes, egg cards, and cotton balls were replaced at 08:00 h every day until the adults died. The total number of eggs laid per day was recorded.

### 2.2. Experimental Protocol

**Figure 1:**
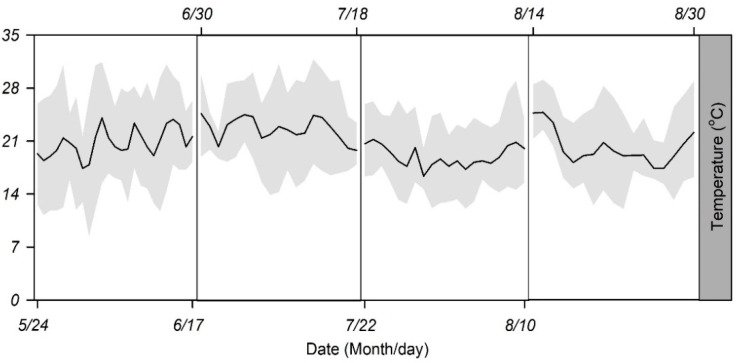
Daily mean temperature (DMT) (solid black line) and daily mean temperature amplitude (DTA) (grey area) experienced by cruciferous vegetable species grown in fields from 24 May to 30 August 2011 in Fanshi County, Shanxi Province, China.

Four batches of new eggs produced within 4 h (approximately 200 eggs per batch) were placed outside the lab to be exposed to different ambient temperatures in Fanshi County, a major cruciferous vegetable planting area in Shanxi Province, China. Here, the mean monthly temperature does not significantly change in summer when vegetables are usually planted; however, the monthly temperature amplitudes change significantly (Table 1 and Figure 1). Batches 1–4 were placed outdoors from 24 May to 17 June, 30 June to 18 July, 22 July to 10 August, and 14 August to 30 August 2011, respectively, and exposed to different daily temperature amplitudes. 

*P. xylostella* were developed outside under the different daily temperature amplitudes from the egg stage to the pupal stage. During this time, the larvae were artificially fed. Then, the newly emerged adults (within 4 h of emergence) were moved indoors (at a constant temperature of 25 ± 1 °C and a moderate RH of 50–70%) and divided into two groups.

In the first group, after pupal emergence, the new females were paired with males in 4 h. The numbers of new pairs for the different temperature amplitudes were as follows: ±4.1 °C: 10; ±5.2 °C: 10; ±6.1 °C: 11; ±7.3 °C: 11. The new pairs were transferred to the glass tubes for mating and oviposition. Then, the longevity (number of days from emergence to death), total fecundity (total number of eggs), and early fecundity (percentage of eggs laid during the first three days of the oviposition period relative to the total number of eggs) were measured.

In the second group, the heat and cold tolerance of each adult was measured using Huber (Peter Huber Kältemaschinenbau AG, Offenburg, Germany). To measure heat tolerance, adults were placed in a closed and transparent reaction kettle (Huber external equipment). The ambient temperature in the reaction kettle was increased from 22 to 45 °C at a rate of 0.1 °C/min. During this period, we recorded heat coma temperature (CT_max_) as the temperature at which the last movement of an individual’s foot or tentacle was observed. Sixty adults were used for each treatment with a sex ratio of 1:1. To measure the cold tolerance, adults were placed in the same equipment. The ambient temperature in the reaction kettle was decreased from 22 °C to 15 °C at a rate of 0.5 °C/min, followed by a decrease to 0 °C at a rate of 0.1 °C/min, and the individuals were maintained at this temperature for 5 min. During this period, we recorded cold coma or chill coma temperature (CT_min_) as the temperature at which the last movement of an adult’s foot or tentacle was observed. Sixty adults per treatment were used with a sex ratio of 1:1.

### 2.3. Statistical Analysis

All data were analyzed using SPSS 21.0 (SPSS Inc., Chicago, IL, USA). All data were evaluated for normality and homoscedasticity and, if needed, were arcsine-, square root-, or log-transformed. The CT_min_ and CT_max_ of males and CT_max_ of females were square root-transformed, whereas the longevity of males and females was transformed using log10 (*x* + 1) before analysis. Pearson correlation coefficient [46] and stepwise multiple regression [47] was performed to determine how the main temperature-related factors, such as the daily mean temperature, daily temperature amplitude, daily high temperature, and daily low temperature, affected different traits of *P. xylostella*. The data were subjected to one-way analysis of variance (ANOVA) to assess the effects of different DTAs on the longevity of females and males, total fecundity, early fecundity, CT_min_, and CT_max_. Means were separated using Tukey’s honestly significant difference (HSD) test (one-way ANOVA) when significant differences were found at *p* < 0.05, and they are shown as mean ± standard deviation (SD). 

## 3. Results

### 3.1. Correlation Analysis among P. xylostella Traits and Temperature Factors

Pearson correlation analysis was performed to explore the possible association among temperature factors and *P. xylostella* traits. The daily mean temperature, daily high temperature, and daily low temperature were not correlated with *P. xylostella traits*. The daily temperature amplitude was highly correlated with most of the traits of *P. xylostella* (*r*^2^ > 0.7) (Table 2). The stepwise multiple regression analysis revealed that the daily mean temperature did not affect *P. xylostella* traits. Daily high temperature, and daily low temperature affected female CT_max_ (*y*_4_ = 5.962 *x*_1_ *−* 5.102 *x*_2_ + 4.433 *x*_3_; *F* = 41.917; df = 3.120; *p* < 0.001) (Table 3). Daily temperature amplitude significantly affected the male longevity (*y*_1_ = −0.441 *x*_1_; *F* = 9.658; df = 1.42; *p* = 0.003), total fecundity (*y*_2_ = −0.479 *x*_1_; *F* = 11.902; df = 1.42; *p* = 0.001), early fecundity (*y*_3_ = −0.497 *x*_1_; *F* = 12.140; df = 1.42; *p* = 0.001), female CT_max_ (*y*_4_ = 5.962 *x*_1_ *−* 5.102 *x*_2_ + 4.433 *x*_3_; *F* = 41.917; df = 3.120; *p* < 0.001), and male CT_max_ (*y*_5_ = 0.658 *x*_1_; *F* = 89.926; df = 1.120; *p* < 0.001) (Table 3). Through these analyses, the most important factor in *P. xylostella* traits was assigned to the daily temperature amplitude.

### 3.2. Influence of Temperature Amplitude on the Longevity and Fecundity

**Figure 2:**
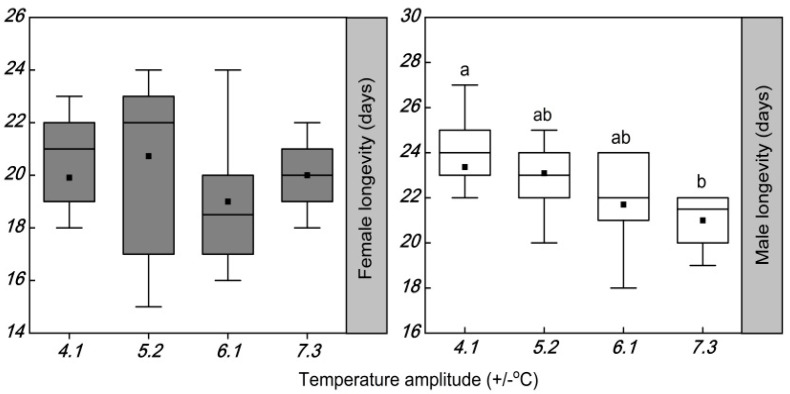
Box plot of the longevity of females and males of *Plutella xylostella* developed under four daily temperature amplitudes, with a similar mean temperature. The upper and lower boundaries of the box indicate the 75th percentile and 25th percentile of the data set. The black horizontal line and black square within the box represent the median and mean values, respectively. Error bars above and below the box indicate the minimum and maximum values, respectively. Different letters above each box indicate significant differences (*p* < 0.05) among the treatments.

The temperature amplitudes had no significant effect on the longevity of females (*F* = 1.081; df = 3, 42; *p* = 0.369), but significantly increased the longevity of males (*F* = 4.309; df = 3, 42; *p* = 0.010) (Table 4 and Figure 2). There were significant differences in the longevity of males among the amplitude temperatures of ±4.1, ±5.2, and ±7.3 °C. The longevity of males was reduced by 0.3, 1.7, and 2.4 days at temperature amplitudes of ±5.2, ±6.1, and ±7.3 °C, respectively, compared with that at the amplitude temperature of ±4.1 °C.

**Figure 3:**
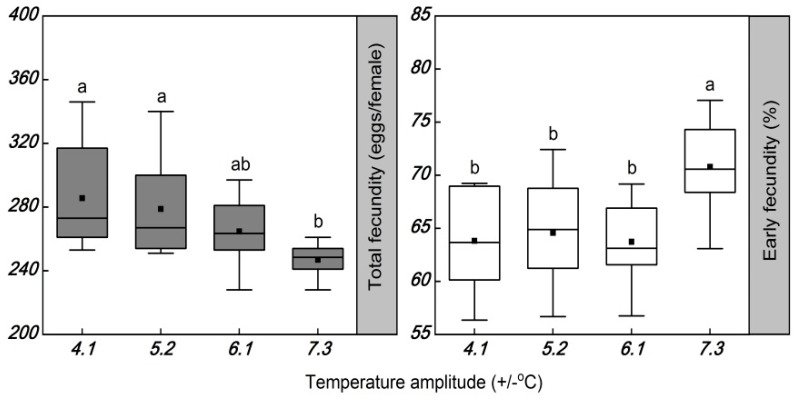
Box plot of the total fecundity and early fecundity in *Plutella xylostella* developed under four daily temperature amplitudes, with a similar mean temperature. The upper and lower boundary of the box indicate the 75th percentile and 25th percentile of the data set. The black horizontal line and black square within the box represent the median and mean values. Error bars above and below the box indicate the minimum and maximum values, respectively. Different letters above each box indicate significant differences (*p* < 0.05) among the treatments.

The temperature amplitudes significantly decreased the total fecundity (*F* = 4.094; df = 3, 42; *p* = 0.013) (Table 4 and Figure 3). The total fecundity was significantly different among females exposed to the temperature amplitudes of ±4.1, ±5.2, and ±7.3 °C. Compared to the temperature amplitude of ±7.3 °C, the total fecundity was increased by 36.0 and 31.9 eggs per female at temperature amplitudes of ±4.1 and ±5.2 °C, respectively.

The temperature amplitudes induced a significant increase in early fecundity (*F* = 6.033; df = 3, 42; *p* = 0.0021) (Table 4 and Figure 3). The early fecundity increased by 7.0%, 6.2%, and 7.1%, in the temperature amplitudes of ±4.1, ±5.2, and ±6.1 °C, respectively, compared with that of females exposed to the temperature amplitude of ±7.3 °C (70.8%).

### 3.3. Influence of Temperature Amplitude on the Thermal Tolerance

**Figure 4:**
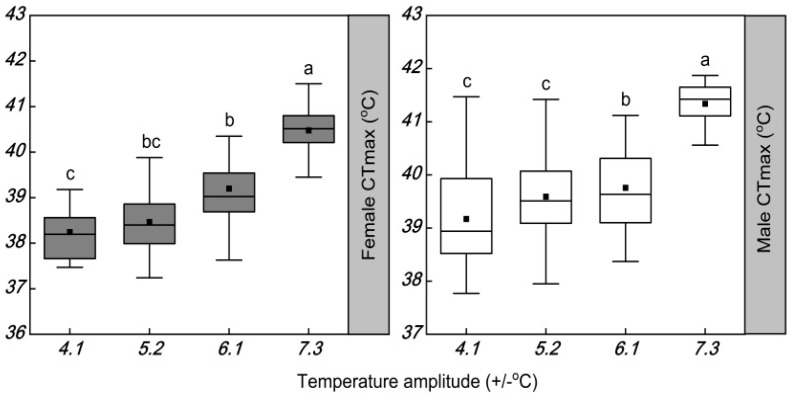
Box plot of the heat coma temperature (CT_max_) of females and males of *Plutella xylostella* developed under four daily temperature amplitudes with a similar mean temperature. The upper and lower boundaries of the box indicate the 75th percentile and 25th percentile of the data set, respectively. The black horizontal line and black square within the box represent the median and mean values, respectively. Error bars above and below the box indicate the minimum and maximum values, respectively. Different letters above each box indicate significant differences (*p* < 0.05) among the treatments.

**Figure 5:**
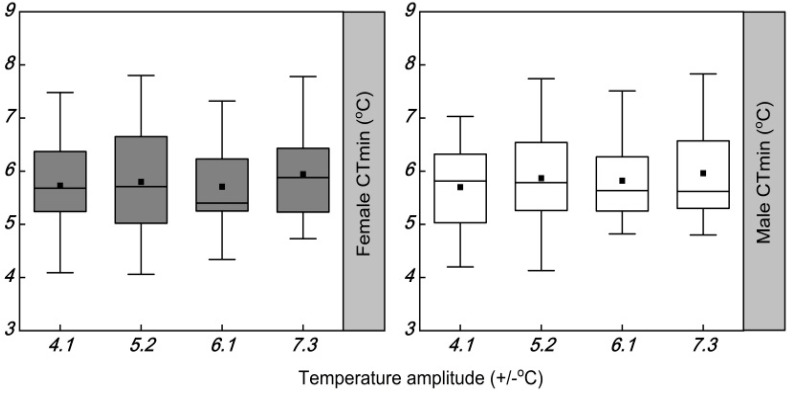
Box plot of the cold coma temperature (CT_min_) of females and males of *Plutella xylostella* developed under four daily temperature amplitudes, with a similar mean temperature. The upper and lower boundaries of the box indicate the 75th percentile and 25th percentile of the data set, respectively. The black horizontal line and black square within the box represent the median and mean values, respectively. Error bars above and below the box indicate the minimum and maximum values, respectively. Different letters above each box indicate significant differences (*p* < 0.05) among the treatments.

The temperature amplitudes significantly increased the CT_max_ of females (*F* = 44.374; df = 3, 120; *p* < 0.001) (Table 4 and Figure 4). The CT_max_ of females was reduced by 2.1, 1.7, and 1.6 °C at temperature amplitudes of ±4.1, ±5.2, and ±6.1 °C, respectively, compared with the widest temperature amplitude of ±7.3 °C. The temperature amplitudes also had a significant increase in the CT_max_ of males (*F* = 53.481; df = 3, 120; *p* < 0.001) (Table 3). The CT_max_ of male was significantly different between the temperature amplitudes of ±7.3 and ±6.1 °C, but not between the temperature amplitudes of ±5.2 and ±4.1 °C. Compared with the temperature amplitude of ±7.3 °C, the temperature amplitudes of ±4.1, ±5.2, and ±6.1 °C caused the CT_max_ of males to decrease by 2.2, 2.0, and 1.3 °C, respectively. The temperature amplitudes had no significant effect on the CT_min_ of females (*F* = 0.502; df = 3, 120; *p* = 0.682) or males (*F* = 0.374; df = 3, 120; *p* = 0.772) (Table 4 and Figure 5).

## 4. Discussion

### 4.1. Influence of Temperature Amplitudes on Longevity and Fecundity

Temperature amplitudes had differential effects on the longevity of adult *P. xylostella*. The amplitudes did not affect the longevity of females, but the longevity of males was significantly reduced with the increase in temperature amplitudes. This difference can be attributed to a higher heat tolerance in females than in males [48]. In addition, with an increase in the temperature amplitudes, when the daily high temperature was close to or exceeded 30 °C, male longevity gradually shortened. This suggests that temperature did not inhibit male longevity above 30 °C. This result differed from that which was previously reported. For example, at constant temperatures, male longevity was 8.9 days at 20 °C; at 27.5 °C, it reached its lowest value (6.7 days), but it was significantly increased up to 9.2 days at 30 °C [49]. This indicates that male longevity was significantly prolonged at 30 °C. This difference could be because when *P. xylostella* individuals are exposed to high daily temperatures, their metabolic rates for the synthesis of heat shock proteins and polyhydroxy compounds increase, and so does their heat resistance [50]. When an adult is exposed to a high temperature during the day, an appropriate recovery temperature at night can slow down the metabolic rate and reduce energy loss, allowing the insect to accumulate sufficient energy and resist the adverse effects of the next high-temperature period. Thus, after exposure to daily high temperatures, the appropriate recovery temperature at night plays a role in the repair of the cellular or protein structural recovery of insect [51].

The temperature amplitude of ±7.3 °C significantly inhibited the total fecundity of *P. xylostella*. Insects subjected to various environmental stresses, such as malnutrition [52], temperature stress [45,53], and oxygen stress [54], at a certain developmental stage can experience direct negative effects on survival, development, and reproduction. These outcomes can be attributed to no significant change in the number of cells in the body after an organism experiences environmental stress at a certain developmental stage [55], but they have a significant effect on energy distribution in the body [56]. When the *P. xylostella* individual was subjected to a temperature amplitude of ±7.3 °C (daily high temperature up to 30 °C), it led to a higher expression of heat shock proteins (HSPs) [57]. This process increases energy consumption [58], which may cause a significant reduction in the total fecundity [59].

We also found that early fecundity by adults significantly increased after exposure to a temperature amplitude of ±7.3 °C. The early fecundity significantly increased (up to 71%) at the temperature amplitude of ±7.3 °C, compared with 64% of females at the temperature amplitude of ±4.1 °C. Environmental stress can induce adults to lay eggs as early as possible to avoid adverse effects later in life [45,60]. In our study, the variation in early fecundity, caused by a daily high temperature (30 °C) with a temperature amplitude of ±7.3 °C, may have been a response to temperature stress, causing accelerated spawning under adverse conditions to maximize fitness before further deterioration and to prevent further damage later in the life cycle, or may be a trade-off of temperature acclimation at the expense of fecundity.

### 4.2. Influence of Temperature Amplitude on Thermal Tolerance

There was a significant difference in the heat tolerance of adults after a change in the temperature amplitudes. However, the cold tolerance of adults was not affected. There are two hypotheses on the relationship between cold tolerance and heat tolerance in insects. One hypothesis suggests that heat and cold tolerances in insects are relatively independent of each other [61], the other hypothesis suggests that there is a positive correlation between heat and cold tolerances in insects [62]. Our results showed that the heat and cold tolerances (CT_max_ and CT_min_) of *P. xylostella* adults were relatively independent of their responses to hot and cold periods in a fluctuating-temperature experiment, suggesting that different regulatory mechanisms or pathways might be involved [13]. We also showed that even when the mean temperature in the natural environment was suitable for the survival and development of *P. xylostella*, its heat tolerance significantly improved when individuals experienced wide temperature amplitudes. This finding is not consistent with previous studies on the heat tolerance of *P. xylostella*. In particular, after feeding at constant temperatures of 20 °C, 25 °C, and 30 °C, no significant differences were observed in the heat tolerance of *P. xylostella* adults [63]. Insects exposed to a high temperature approaching or reaching the heat tolerance limit during a short period produced more heat shock proteins than those exposed to constant temperatures. Wide temperature amplitudes could have beneficial effects on the heat tolerance of insects [64].

### 4.3. Implications for Pest Population Modeling

To understand the effects of environmental conditions on insect population dynamics and distribution, the degree-day model based on the mean temperature was widely used [65,66,67]. However, the effects of daily temperature amplitude at similar mean temperatures are often overlooked. We found that the longevity, total fecundity, early fecundity, and thermal tolerance of *P. xylostella* significantly changed under DTAs. These temperature amplitude patterns during the day and night under similar mean temperatures should be incorporated into prediction models for insect populations to simulate the effects of complex temperature patterns in nature on the ecology of *P. xylostella* populations and to predict the occurrence of this pest in the field.

## 5. Conclusions

In summary, this study provides an overview of the longevity, total fecundity, early fecundity, and thermal tolerance of *P. xylostella* under temperature fluctuations in a field setting. It is imperative to employ temperature fluctuations corresponding to the field conditions experienced by *P. xylostella* from the egg stage to the pupal stage. Our findings highlight the effects of DTAs on the phenotype and acclimation response of *P. xylostella*, thus emphasizing the importance of considering DTA when predicting models for assessing insect populations and the effects of climate change.

## Figures and Tables

**Table 1:** Actual recorded ambient temperatures (mean ± SD) in different treatment groups.

Group	Duration	Actual Recorded Temperature			
		DMT ^1^ (°C)	DTA (±°C)	DHT (°C)	DLT (°C)
1	5/24–6/17	20.8 ± 1.8 b	7.3 ± 1.7 a	27.6 ± 2.3 a	14.2 ± 2.6 b
2	6/30–7/18	22.6 ± 1.5 a	5.2 ± 1.6 ab	28.0 ± 2.6 a	17.3 ± 1.9 a
3	7/22–8/10	22.8 ± 1.6 a	6.1 ± 1.2 ab	29.3 ± 2.4 a	17.3 ± 1.9 a
4	8/14–8/30	20.2 ± 2.3 b	4.1 ± 1.4 b	25.4 ± 2.7 b	16.0 ± 3.0 ab
	df	3.25	3.19	3.20	3.17
	MS	33.012	38.271	47.493	51.252
	*F*	9.809	16.893	7.658	9.120
	*p*	<0.001	<0.001	<0.001	<0.001

^1^ DMT, DTA, DHT, and DLT represent the daily mean temperature, daily mean temperature amplitude, daily mean high temperature, and daily mean low temperature, respectively. Different letters indicate significant differences (*p* < 0.05) among the treatments with Tukey HSD test.

**Table 2:** Results of the Pearson correlation coefficient among *Plutella xylostella* traits and temperature factors.

Trait	DMT (°C)	DTA (±°C)	DHT (°C)	DLT (°C)
Female longevity	−0.12	−0.22	−0.35	−0.09
Male longevity	−0.07	−0.96 * ^1^	−0.56	+0.52
Total fecundity	+0.07	−0.97 *	−0.43	+0.66
Early fecundity	−0.39	+0.79	+0.02	−0.88
Female CT_max_	−0.20	+0.92	+0.30	−0.71
Male CT_max_	−0.13	+0.96 *	+0.51	−0.46
Female CT_min_	−0.32	+0.70	+0.01	−0.80
Male CT_min_	+0.24	+0.90	+0.54	−0.42

^1^ Significant *p*-values are indicated in a bold typeface. * represents a significant difference at *p* < 0.05.

**Table 3:** Stepwise multiple regression with Plutella xylostella traits as the dependent variable and temperature factors as independent variables.

Mode	df	MS	*F*	*p*
*y*_1_ ^1^ = −0.441 *x*_1_^2^	1.42	36.823	9.658	**0.003** ^3^
*y*_2_ = −0.479 *x*_1_	1.42	7751.408	11.902	**0.001**
*y*_3_ = −0.497 *x*_1_	1.42	0.027	12.140	**0.001**
*y*_4_ = 5.962 *x*_1_ − 5.102 *x*_2_ + 4.433 *x*_3_	3.120	24.755	41.917	**<0.001**
*y*_5_ = 0.658 *x*_1_	1.120	62.018	89.926	**<0.001**

^1^*y*_1_, *y*_2_, *y*_3_, *y*_4_, and *y*_5_ represent, respectively, male longevity, total fecundity, early fecundity, female CT_max_, and male CT_max_. ^2^ *x*_1_, *x*_2_, and *x*_3_ represent, respectively, daily temperature amplitude, daily high temperature, and daily low temperature. ^3^ Significant *p*-values are indicated in a bold typeface.

**Table 4:** Effects of the daily temperature amplitude (DTA) on female and male *Plutella xylostella* traits as determined by ANOVA.

Trait	df	MS	*F*	*p*
Female longevity	3.42	0.071	1.081	0.369
Male longevity	3.42	0.118	4.309	**0.010** **^1^**
Total fecundity	3.42	2752.413	4.094	**0.013**
Early fecundity	3.42	116.564	6.033	**0.002**
Female CT_max_	3.120	0.165	44.374	**<0.001**
Male CT_max_	3.120	30.194	53.481	**<0.001**
Female CT_min_	3.120	0.015	0.502	0.682
Male CT_min_	3.120	0.341	0.374	0.772

^1^ Significant *p*-values are indicated in a bold typeface.

## Data Availability

The data used to support the findings of this study are available from the corresponding author upon request.
